# Implementation of a Home Monitoring System for Heart Failure Patients: A Feasibility Study

**DOI:** 10.2196/resprot.5744

**Published:** 2017-03-20

**Authors:** Martin Steven Kohn, Jeffrey Haggard, Jack Kreindler, Kade Birkeland, Ilan Kedan, Raymond Zimmer, Raj Khandwalla

**Affiliations:** ^1^ Sentrian San Juan Capistrano, CA United States; ^2^ Cedars-Sinai Heart Institute Beverly Hills, CA United States

**Keywords:** heart failure, home monitoring, predictive analytics, patient engagement

## Abstract

**Background:**

Improving the management of patients with complex chronic disease is a substantial undertaking with the simultaneous goals of improving patient outcomes and controlling costs. Reducing avoidable hospitalization for such patients is a step toward both objectives. Some of the deterioration experienced in chronic disease patients occurs outside the view of their clinicians, and before the patient becomes overtly symptomatic. Home monitoring has been used for more than 20 years to detect deterioration earlier so that the patients could be treated before they became ill enough to require hospitalization. Patient participation is an important requirement for successful home monitoring. There has been some concern that patients would be unwilling or unable to engage in a program that collected multiple measurements. The Cedars-Sinai Cardiology Center provides a high-touch, intense management program for patients with congestive heart failure (CHF). A group of their patients were chosen to join a complex, multidevice home monitoring system to see whether such patients would find value in the additional effort.

**Objective:**

The objective of our study was to determine whether patients already actively engaged in a high-touch intensive management program for CHF would take on the additional burden of a complex home monitoring effort.

**Methods:**

A total of 20 patients from the Cedars-Sinai group were enrolled in a monitoring program utilizing 5 different devices. Anonymous surveys were collected from the patients to assess their satisfaction with the program.

**Results:**

In total, 90% (18/20) completed the program, and 61% (11/20) submitted the survey. Among the 18 patients, overall compliance with the requested measurements was 70%. It was found that 73% (8/11) felt better about their health as a result of the program, whereas another 73% (8/11) believed that the care team now had a better picture of their health.

**Conclusions:**

Substantial patient compliance and satisfaction can be achieved in a sophisticated home monitoring program.

## Introduction

The management of chronic disease is a substantial burden, both for the patients and the provider organizations supporting them. In total, 71% of health care expenditures in the United States result from patients with multiple chronic diseases such as congestive heart failure (CHF), chronic obstructive pulmonary disease (COPD), and diabetes [1]. A substantial fraction of the cost is generated by repeated hospitalization and emergency department (ED) visits [1]. The incidence of chronic disease continues to grow, in part because of an aging population and improved management of chronic diseases. Better acute coronary care, with a 38% decrease in the death rate from coronary artery disease in the last decade [2], results in more patients surviving and living with CHF. Further, changes in the social environment have resulted in more seniors living independently, often at a distance from children who might be monitoring their well-being. One of the strategies for controlling the cost of health care, while simultaneously improving outcomes and quality of life for patients, is to reduce avoidable hospitalization for patients with chronic diseases. CHF, COPD, and diabetes are included in the list of ambulatory care sensitive diseases, as defined by the Agency for Healthcare Research and Quality (AHRQ), meaning improved outpatient care can reduce the need for hospitalization [3].

Identifying patients early in the course of decompensation provides the opportunity for a modest intervention to reduce the likelihood of hospitalization. Since patients spend most of their lives without direct communication with their clinical care team, early deterioration can be missed. There have been attempts to close that communication gap with “high-touch” programs involving frequent phone contact or home visits to improve outpatient follow-up and evaluation. Many of these programs have been effective in reducing the number of hospitalizations, but have been hampered by their labor-intensive, high-cost structure, which has made it difficult to scale the programs beyond a small number of patients [4-7].

Over the last 20-30 years, home monitoring devices have been used to make outpatient evaluation more effective. Some such efforts resulted in a reduction in hospitalizations, but were plagued with false alarms and missed opportunities [4,5], whereas others reported no benefit [6,7].

Patient participation has been a challenge. Many of the earlier devices either required patients to actively take measurements, such as blood pressure or weight, and record it in a log or submit the result by phone [8]. Reports on patient compliance in monitoring programs are mixed. Patient adherence to the monitoring protocol was often good when only a small number of measurements with a small number of devices were required [9-11]. However, others reported poor compliance [12,13]. The inefficiency of the process, and demands on the patient, were disincentives to participation. Devices that automatically recorded and transmitted the readings became available, but were expensive and still required substantial effort on the part of the patient. One group reported high compliance in telephone mediated reporting of multiple measures, after specifically excluding patients for “poor compliance with HT systems” [8]. Another report, also working with exclusion criteria, reported a sharp decrease in compliance over 6 months of the study [6]. It may be appropriate to treat remote monitoring as you would any other intervention, namely, selecting patients who are more likely to benefit. Financial incentives provided little long-term improvement in participation, with modest compliance in the incentivized groups and poor compliance in the unincentivized group [14].

Fortunately, device technology has evolved rapidly, making it easier to safely and securely collect and transmit data [15]. This allows us to work with multiple data streams, rather than just one or two. New measures previously not readily available, such as oxygen concentration or intrathoracic fluid assessment increase opportunity to incorporate novel data streams. However, just collecting data has relatively little value. The expanded interest in and availability of patient generated health data (PGHD), including patient-reported outcomes, means we need to better understand patient willingness to participate in more complex monitoring programs [16]. The data has to be analyzed to provide actionable insights used by decision makers, be they the patient, the patient’s family or the clinicians, to help make better decisions. Improvement in analytic techniques allows us to find data patterns with strong predictive power, well beyond that obtained by having someone “eyeball” the collected data. Humans rely on experience and intuition, which cannot effectively deal with the volume and complexity of big data [17].

For most patients with diseases such as CHF and COPD, the processes that lead to severe symptomatic acute illness develop over time, sometimes weeks before the patient presentation or clinical discovery. Even though the patient may not overtly recognize that illness is progressing, seeking patterns in physiologic data such as heart rate, heart rate variability, oxygen concentration, and blood pressure could allow the early detection of those processes. With earlier detection, there may be opportunities for prevention of progression to symptoms and clinical decompensation in advance of severe acute illness that might require hospitalization or other major intervention.

Target outcomes of modern monitoring technology with predictive analytics are reduced hospitalizations, ED visits, or unscheduled office visits, compared with a baseline of such events in the absence of monitoring. Concomitants for obtaining benefit from the early warning provided by the predictive analytics are a system that promotes patient participation and a care management program that can respond effectively to the notifications and track the events. Given the reports of limited patient participation in systems using a small number of devices [9-11], we want to determine if patients already engaged in a high-touch, effective treatment program will participate in a complex home monitoring effort using multiple devices.

This report describes the WEAR-HeFT (Wearable Device Monitoring Heart Failure) trial, which is a pilot program designed to address all the requirements of an effective home monitoring program for patients with late stage CHF. The Cedars Sinai Medical Group in Los Angeles, CA has implemented the Heart Failure Drug Therapy Management Program to improve outcomes and reduce hospitalization. It targeted patients with high levels of hospitalization and utilization of acute care services. The program was effective in that it reduced both CHF and all cause admissions by 50% compared with a 36% reduction in a control group [18].

Sentrian Remote Patient Intelligence is a commercial predictive analytic, machine-learning platform created to provide the data management and predictive analytics necessary to process home monitoring data. The goal is to identify patterns in the data that suggest early deterioration and generate the notification that a patient will become acutely ill some days hence. The Sentrian platform is device and data stream agnostic. It can, for example, utilize data from home monitoring devices such as oxygen saturation, blood pressure, or temperature. It can also work with laboratory results such as natriuretic peptide, activity, sleep quality, and patient reports of matters (eg, pain and anxiety). The Sentrian system allows the clinicians to create rules to analyze the data streams over both short and long-term trends to generate notifications when specified conditions are met. The rules are designed to be appropriate for the individual patient. The accuracy of predictions about deterioration is one of the sources of feedback to support machine learning to improve predictive power. Health care organizations are charged a monthly fee for each monitored patient.

The pilot WEAR-HeFT trial is designed to demonstrate whether patients who are already engaged in an intensive outpatient program for CHF will participate in a complex home monitoring program. Successful patient engagement is a predicate for further study to assess the predictive power of the analytic platform.

This report specifically addresses lessons learned about the implementation process to enroll patients in a monitoring program, teach them the use of the devices, and overcome obstacles, both anticipated and unanticipated.

## Methods

### Inclusion and Exclusion Criteria

Patients for the program were chosen by the staff at Cedars-Sinai Medical Center from the group that was enrolled in the Heart Failure Drug Therapy Management Program. Specific inclusion and exclusion criteria are listed in Textboxes 1 and 2. The project was reviewed and approved by the Cedars-Sinai Institutional Review Board.

**Inclusion criteria for the study.**

Inclusion criteria

- Patient with New York Heart Association (NYHA) Class 2-4
- Brain natriuretic peptide (BNP) greater than 150pg/mL
- History of heart failure admission at least one time in the previous 12 months or requiring frequent outpatient follow-up (repeat visits on a monthly or weekly basis)
- Competent to give informed consent
- Deemed to be good candidates for the study by investigators

**Exclusion criteria for the study.**

Exclusion criteria

- Unable to give informed consent or understand research protocol
- Immobile
- Physically unable to wear devices
- History of missing clinic appointments
- Unwilling to complete follow up evaluation
- Unstable psychiatric illness
- End stage renal disease (CrCl <15mL/min)
- Recent history of pneumonia

### Safety Parameters

A set of devices was chosen for each patient to provide the desired measurements. The devices approved by Food and Drug Administration (FDA) were the ForaCare W310B Weight scale, ForaCare D40D Blood Pressure Monitor, ChoiceMed MD300C318T2 Pulse Oximeter, and the CoVa from ToSense for intrathoracic fluid. Additionally, the Fitbit Flex (Fitbit, San Francisco, CA) was used for tracking numbers of steps. Qualcomm’s 2Net Hub was used to link the devices to the cloud in a Health Insurance Portability and Accountability Act (HIPAA) compliant environment. Potential participants in the program met with the cardiology staff and had the details of the program explained to them, including potential benefit and risks. The Fitbit Flex was worn continuously, whereas the other measurements (weight, blood pressure, pulse oximeter, CoVa) were performed once a day first thing in the morning. Pulse rate was detected from the oximeter. Patients were asked if they had wireless Internet access since such access was required for data transmission and acquisition. Those that agreed to participate were given instruction in the use of the devices and provided a phone number to call for any additional instruction or troubleshooting. All participants signed an approved informed consent document. Patients were enrolled on a gradual basis and duration of participation ranged from 40 to 117 days.

The ultimate purpose of Sentrian’s analytics is to predict deterioration in advance and not as an alarm for critical conditions. However, the Cedars team established criteria as a fail-safe for which a cardiologist would be notified immediately, shown in Textbox 3. The Cedars investigators were kept blinded with respect to the data. An independent cardiologist was notified when the safety parameters were exceeded.

**Safety parameters.**

- Systolic blood pressure (SBP) >180mmHg
- Diastolic blood pressure (DBP) <50mmHg
- Heart rate (HR) >150bpm
- HR <50bpm
- Pulse oximetry <90%
- Weight gain 5 lb in 24 h
- Weight loss 5 lb in 24 h

Several issues arose during the early stages of the project. First, despite what were thought to be careful explanations there was a misunderstanding of wireless and Internet connections among the patients. Several patients claimed they had wireless access when they did not and thus had to be provided with an Internet hot spot (Mifi) to participate. Most of the patients had difficulty with the finger dexterity necessary to close the clasp on the Fitbit. An alcohol-based hand sanitizer was used as a short-acting lubricant to facilitate clasp closure.

All patients were asked to evaluate the program through an anonymous survey at the conclusion of the program. Each question was answered by marking the desired response: strongly agree, agree, neutral, disagree, and strongly disagree. The survey was completed on paper with the patients circling one of the responses to each question. Textbox 4 provides the items included in the survey.

**Items included in the survey.**

- I feel better connected to my Care Team.
- The time spent taking measurements is worthwhile.
- I feel better about my health.
- The Technical Assistance has been very helpful.
- It is easy to contact Technical Support, if needed.
- The program provides a more complete picture of my health to my provider.
- My Care Team has been responsive and helpful.
- I have been compliant taking my measurements.
- I take my measurements at the same time each day.
- If I forget to take my measurements, a reminder would be helpful.

We also tracked compliance with the measurement regimen as the fraction of expected measurement received.

## Results

### Patient Compliance

A total of 20 patients were enrolled in this pilot clinical trial; of which, 2 patients, numbers 4 and 13, dropped out and 18 completed the study, which ended on February 10, 2015. At that time, all data were collected for analysis and reporting. Patient compliance with the measurement schedule is shown in Table 1. The rate of compliance with measurements (fraction of expected measurements received) in the 18 patients who completed the study was 70%. There is a clear demarcation between patients that were actively engaged and those that were not. Five of the patients, including the 2 that dropped, were compliant less than 36% of the time. The minimum compliance in the more active group was 55%. Compliance in the active group was 78%. Patients that dropped out or stopped collecting data gave several explanations, including a too complex regimen, frustration with the devices, inability to complete the measurement protocol (too sick or too busy), or physical limitations on completing measurements (inability to balance on weight scale). The compliance experience emphasizes the importance of predicting which patients are more likely to actively engage in the program, as noncompliant patients get no value for the cost of the program. The desired clinical value and economic efficiency are most readily achieved if we learn enough about patients who will not participate to focus on those who will. We also need to learn more about the personalized support that may be needed to keep patients actively engaged.

**Table 1:** Patient compliance.

Patient number^a^	Rate of compliance (%)
1	95
2	36
3	83
4	23
5	96
6	89
7	82
8	46
9	71
10	77
11	68
12	57
13	0
14	55
15	35
16	95
17	89
18	76
19	58
20	76

^a^Patients 4 and 13 dropped out.

### Survey for Patient Satisfaction

**Figure 1:**
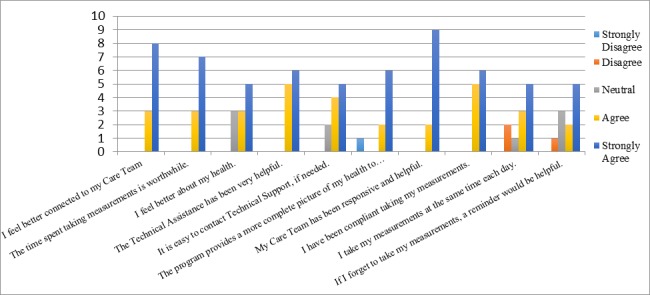
Patient satisfaction survey.

The results of the patient satisfaction survey are shown in Table 2 and displayed graphically in Figure 1. Eleven patients (61%) completed the satisfaction survey. Since the survey was anonymous, we could not determine why the others did not respond. Two patients did not answer all the questions. All patients either agreed or strongly agreed that they felt better connected to their care teams and that taking the measurements was worthwhile. It was found that 8 of the 11 patients (73%) felt better about their health as a result of the program, whereas another 8 of 11 (73%) believed that the care team now had a better picture of their health.

**Table 2:** Patient satisfaction survey.

Item	Strongly disagree	Disagree	Neutral	Agree	Strongly agree	Did not answer
I feel better connected to my Care Team.	0	0	0	3	8	0
The time spent taking measurements is worthwhile.	0	0	0	3	7	1
I feel better about my health.	0	0	3	3	5	0
The Technical Assistance has been very helpful.	0	0	0	5	6	0
It is easy to contact Technical Support, if needed.	0	0	2	4	5	0
The program provides a more complete picture of my health to my provider.	1	0	0	2	6	2
My Care Team has been responsive and helpful.	0	0	0	2	9	0
I have been compliant taking my measurements.	0	0	0	5	6	0
I take my measurements at the same time each day.	0	2	1	3	5	0
If I forget to take my measurements, a reminder would be helpful.	0	1	3	2	5	0

A chi-square contingency table analysis was performed on the response data in Table 2, using Microsoft Excel 2016, version 16.0.6965.2115. It did not quite reach statistical signification, with a *P* value of .12, in part due to lack of independence among the responses. A positive response to one question was likely associated with a positive response to another question.

## Discussion

### Principal Findings

Several issues arose during the early stages of the project. First, despite what were thought to be careful explanations there was a misunderstanding of wireless and Internet connections among the patients. Several patients claimed they had wireless access when they did not and thus had to be provided with an Internet hot spot (Mifi) to participate. Many of the patients had difficulty with the finger dexterity necessary to close the clasp on the Fitbit. An alcohol-based hand sanitizer was used as a short-acting lubricant to facilitate clasp closure.

A few of the patients relied on a wheeled walker for ambulation. Weight measurements for those patients were unreliable as they were affected by the variations in level of support that each patient needed from the walker. Although we intentionally limited the number of devices requiring active participation by the patients, a few patients initially felt that the measurement process was too complex. The problem seemed to diminish as the patients became more accustomed to the process. Some of the patients had substantially healthier spouses or significant others that provided support and helped the patients with the measurement process. On one hand, there was an advantage to the help provided. However, sometimes the spouse answered questions for the patients or dominated the discussion so that it was difficult to ascertain the patient’s level of understanding. It became clear that special care was necessary to ensure that both the patient and the care-giver had the same understanding.

Some of the participants had already been using home monitoring devices such as blood pressure cuffs and weight scales. Some patients were concerned by the different readings from the new devices provided for the study. The team explained that such differences were minor and expected and were not alarming and that data trends were more important. Although each patient was given personal instruction in the use of devices, most benefited from phone support when setting up the monitors. There were several cases of idiosyncratic behaviors, with some patients calling technical support or not wanting to use a particular device, requiring additional support.

Many previous reports on compliance in home monitoring involved the use of one or two devices. We added a regimen with multiple devices to an existing intense, management program that already placed heavy demands upon the patients. We have shown that home monitoring produces additional value in such a comprehensive environment. Compliance in our group was at least comparable with compliance reported in other studies, confirming that a complex home monitoring regimen is feasible [10,11,14,15].

There was a high level of satisfaction among the patients, with strong feelings that the program improved their comfort with their health and left them more connected with their health care team. The sharp demarcation between patients that were either poorly compliant with the measurement schedule or dropped out of the program emphasized the need for a personalized approach to home monitoring. Despite a robust implementation and training program, some patients stopped taking the measurements. Distinguishing between patients who will participate if given extra support from those who will not engage is an important part of implementing a clinically and economically valuable program.

### Limitations

This was a small study with a group of patients chosen who already had a close relationship with their care providers. The patients were chosen by the staff cardiologists to include patients that had been heavy utilizers of acute health services. However, we cannot exclude bias in that selection process, which might affect the results. It may not be generalizable to a broader group of patients with less tight ties. Also, 7 of the 18 patients did not respond to the survey. It is possible that all of those patients found little value in the program. If so, it would still leave a majority of the patients finding value. Despite the preexisting strong association with the cardiology team, many patients felt that the monitoring program added to the relationship. Since this was a feasibility study with no control group, no comparative statistical analysis was possible. Since the survey was anonymous, it was not possible to relate the responses to individual patient characteristics, such as time in the program, which could have biased the result.

### Conclusions

A carefully designed, home monitoring pilot implementation program using commercially available wearable devices in an insured elderly CHF cohort demonstrates feasibility in using a multimodality home monitoring strategy for selected patients. The study validated that patients can work with multiple devices, providing an array of data streams, as well as insights into unexpected challenges. Most obstacles to patient engagement can be overcome with appropriate support and encouragement. Cooperation between the clinical and technical teams is the key, as is identifying patients more likely to benefit from the program, those that need extra support, and those who will find no value in it.

Future directions would include expanding to larger numbers of patients with multiple chronic conditions, identifying which combinations of devices and data streams are most helpful for particular patients and refining impactibility, which patients are likely to benefit from home monitoring.

## Abbreviations

AHRQ: Agency for Healthcare Research and Quality
BNP: Brain natriuretic peptide
CHF: congestive heart failure
COPD: chronic obstructive pulmonary disease
DBP: diastolic blood pressure (DBP)
ED: emergency department
FDA: Food and Drug Administration
HIPAA: Health Insurance Portability and Accountability Act
HR: heart rate
NYHA: New York Heart Association
PGHD: patient generated health data
SBP: systolic blood pressure (SBP)
WEAR-HeFT: Wearable Device Monitoring Heart Failure
